# A Retrospective Study of 10 Cases of Laparoscopic and Laparotomic Risk-Reducing Salpingo-Oophorectomy Performed on Patients With BRCA-Positive Breast Cancer

**DOI:** 10.7759/cureus.78732

**Published:** 2025-02-08

**Authors:** Hiroaki Ishida, Megumi Manrai, Hiroki Egashira, Kouta Arakawa, Akiko Takashima

**Affiliations:** 1 Obstetrics and Gynecology, Toho University Medical Center Sakura, Sakura, JPN; 2 Clinical Laboratory, Toho University Medical Center Sakura, Chiba, JPN

**Keywords:** advanced ovarian cancer, hereditary breast and ovarian cancer, laparoscope, rrso, stic

## Abstract

Introduction: Women who carry the breast cancer gene (*BRCA*)*1/2* pathogenic variants have a higher lifetime risk of developing ovarian cancer than the general population (*BRCA1*, 44%; *BRCA2*, 17%). There is currently no reliable method for the early detection of ovarian cancer, and the prognosis of advanced ovarian cancer is poor. Therefore, risk-reducing salpingo-oophorectomy (RRSO) is recommended for patients with breast cancer who carry *BRCA1/2* pathogenic variants. We retrospectively reviewed 10 cases of RRSO in such patients performed at our hospital.

Methods and results: This study included 10 patients with *BRCA*-positive breast cancer who underwent RRSO after genetic counseling between April 2021 and December 2024. The patients ranged from 39 to 72 years of age (median, 43.5 years), and of the six premenopausal patients, three had symptoms of menopause requiring medication. The surgery types were as follows: laparoscopic surgery (n = 8), laparotomy (n = 1), and conversion from laparoscopy to laparotomy (n = 1). The operative times (median) were as follows: laparoscopy, 59-91 min (85 min), and laparotomy, 76-118 min (97 min). Postoperative histopathological testing revealed no cases of occult cancer or serous tubal intraepithelial carcinoma.

Conclusion: It is difficult to observe the upper abdomen in laparotomic RRSO, whereas laparoscopy allows for visualization of the entire abdominal cavity and a shorter operative time; therefore, laparoscopic surgery is considered a viable option. Post-RRSO patient management requires follow-up to monitor for the development of peritoneal cancer, and in premenopausal women in particular, treatment and follow-up for any symptoms of menopause are needed; therefore, individualized care is required.

## Introduction

Approximately 12,738 women were diagnosed with ovarian cancer in Japan in 2020, and 5,182 women died of ovarian cancer in 2022, making it the gynecological malignancy with the poorest prognosis [1]. Hereditary breast and ovarian cancer syndrome (HBOC) is a syndrome in which individuals with pathogenic variants of breast cancer gene (*BRCA*) *1* or *2* are at a higher risk of developing breast and/or ovarian cancer, among others [2]. *BRCA* is an important gene related to genetic mutations that increase the risk of breast and ovarian cancer. Specifically, there are two genes, *BRCA1* and *BRCA2*, and mutations in these genes increase the risk of developing breast and ovarian cancer. The* BRCA* gene is involved in repairing DNA in cells, and when it functions normally, it works to suppress the risk of cancer, but when it is mutated, this function is impaired. Mutations in the *BRCA *genes are associated with family history and are important indicators in assessing inherited cancer risk. Pathogenic variants are responsible for approximately 13% to 18% of ovarian cancer cases [3,4].

Risk-reducing salpingo-oophorectomy (RRSO) is the most effective measure for preventing ovarian cancer in patients with HBOC, reducing the risk of developing ovarian cancer and breast cancer by approximately 80% and 50%, respectively, as well as reducing the overall risk of death [5-7]. As there is currently no established screening method for ovarian cancer, RRSO is the most effective preventive treatment. RRSO has been available in Japan as an insurance-covered medical treatment for HBOC in patients with breast cancer since April 2020, and our hospital started performing RRSO in April 2021.

No studies have compared laparoscopy with laparotomy RRSO. A study comparing laparoscopy and laparotomy for benign ovarian tumors reported that laparoscopy was superior to laparotomy because it caused less fever and pain and shortened hospital stays by about three days [8]. Since most RRSO procedures involve the removal of the normal adnexa, we believe it would be appropriate to perform the procedure via laparoscopy, similar to that for benign ovarian tumors. 

Risk-reducing salpingo-oophorectomy is currently recommended for patients with breast cancer between 35 and 40 years of age for those with a *BRCA1 *pathogenic variant and between 40 and 45 years for those with a* BRCA2* pathogenic variant [9]. There are concerns, however, that removing both ovaries may lead to symptoms of menopause in premenopausal women, as well as a reduction in bone density. Additionally, the risk of developing peritoneal cancer is higher in cases in which serous tubal intraepithelial carcinoma (STIC), the foundation for the development of ovarian cancer, is detected in excised specimens [10]. Therefore, it is necessary to consider how comprehensive post-RRSO patient management should be carried out, based on the guidelines for ovarian cancer, gynecological care for premature menopause, and HBOC.

In this study, we retrospectively examined cases of RRSO performed at our hospital and discussed appropriate postoperative patient management (follow-up intervals, necessary examinations, and treatment of menopausal symptoms) based on relevant literature. In addition, there have been no studies comparing laparoscopy and laparotomy regarding RRSO. In this study, although the number of cases was small, RRSO was performed by laparotomy, and laparoscopy and laparotomy were compared.

## Materials and methods

This study included 10 patients with* BRCA*-positive breast cancer who underwent RRSO between April 2021 and December 2024. The patient selection criteria were set as follows: 1）Breast cancer with *BRCA1/2* pathogenic variant; 2) 35 years of age or older; 3) No desire to have children; 4) Received adequate genetic counseling; and 5) No serious complications. The surgical procedure was based on the 2020 National Comprehensive Cancer Network (NCCN) guidelines [11]. In principle, RRSO is performed laparoscopically at our hospital. For cases with complications that prevent general anesthesia, we perform RRSO with laparotomy using a combination of spinal and epidural anesthesia. RRSO, the characteristics of which are shown in Table 1, differs from standard salpingo-oophorectomy [11]. Histopathological evaluations of the resected specimens were performed as described in the Sectioning and Extensively Examining the Fimbriated End of the Fallopian Tube (SEE-FIM) protocol proposed by the College of American Pathologists [12]. Medical records were retrospectively reviewed to obtain the following data: patient age, *BRCA* variant, RRSO operative time, operative blood loss, length of hospital stay, presence or absence of complications, presence or absence of occult cancer and/or tubal intraepithelial carcinoma in the resected specimen, presence or absence of postoperative menopausal symptoms, presence or absence of postoperative peritoneal cancer, and presence or absence of prophylactic mastectomy. We also considered appropriate post-RRSO management methods based on a literature review. The protocol for this study was approved by the ethics committee of the Toho University Medical Center Sakura, Sakura, Japan (approval no. S24049) and was performed in accordance with the Declaration of Helsinki. Research information was disclosed and the opportunity to opt out was guaranteed.

**Table 1:** Summary of the RRSO procedure based on the 2020 NCCN guidelines RRSO, risk-reducing salpingo-oophorectomy; NCCN, National Comprehensive Cancer Source: [11]

	Differs from standard salpingo-oophorectomy
1	In principle, RRSO is performed laparoscopically
2	Thoroughly visualize the abdominal cavity
3	Biopsy all abnormal peritoneal lesions
4	Perform peritoneal fluid washing cytology
5	Separate the pelvic infundibulum ligament from the ovaries and fallopian tubes by at least 2 cm
6	Remove fallopian tubes up to the uterine horns
7	Remove any peritoneum overlying the ovaries and fallopian tubes, including adhesions between the ovaries and the pelvic sidewall
8	Use a collection bag to collect the excised specimen

## Results

Table 2 shows the patient background and surgical details of the 10 RRSO surgeries performed at our hospital. Ten patients were between 39 and 72 years old (median 43.5 years) and had four cases of *BRCA1 *and six cases of *BRCA2*. Eight laparoscopic surgeries were performed, and one laparoscopic surgery was performed each for laparotomy and primary laparotomy. Operative time and length of hospital stay tended to be shorter laparoscopically than laparoscopic surgery. In addition, laparoscopic surgery tended to bleed less. There were no cases of STIC, latent cancer, or malignant ascites cells. Of the six premenopausal women in RRSO, three had menopausal symptoms and required drug intervention. Detailed clinical information for 10 patients is shown in Table 3.

**Table 2:** Background of RRSO RRSO: risk-reducing salpingo-oophorectomy; *BRCA:* breast cancer gene; STIC: serous tubal intraepithelial carcinoma (STIC) is a precursor of high-grade serous; RRM: risk-reducing mastectomy

Variables	
Age range (Median), years	39-72 (43.5）
*BRCA *status	*BRCA1*: 4cases
	*BRCA2*: 6cases
Surgical method	Laparoscopy: 8 cases
	Laparoscopy → Laparotomy: 1 case
	Laparotomy: 1 case
Operation time range (median), minutes	Laparoscopy (n=8): 59ｰ91 (85)
	Laparotomy (n=2): 76-118 (97)
Amount of bleeding (ml) range (median)	Laparoscopy (n=8): 5ｰ53 ml (7.5 ml)
	Laparotomy (n=2): 12-124 ml (68 ml)
Postoperative hospital stay (days)	Laparoscopy: 4 days
	Laparotomy: 6 days
Surgical complications	－
Ascites cytology	－
STIC	－
RRM	3 cases
Menopausal symptoms after RRSO	3 cases（39 years, 41 years, 44 years）

**Table 3:** Summary of 10 cases of RRSO performed at our hospital RRSO: risk-reducing salpingo-oophorectomy; *BRCA*: breast cancer gene; STIC: serous tubal intraepithelial carcinoma; RRM: risk-reducing mastectomy

Case	Age (years)	*BRCA *status	Operative (min)	Amount of bleeding (mL)	Postoperative hospital stay (days)	Ascites cytology	STIC	Surgical method	Other
Case 1	56	BRCA1	89	53	4	Negative	Negative	Laparoscopy	RRM
Case 2	64	BRCA1	84	20	4	Negative	Negative	Laparoscopy	
Case 3	44	BRCA2	86	10	4	Negative	Negative	Laparoscopy	RRM menopausal symptoms
Case 4	42	BRCA1	59	5	4	Negative	Negative	Laparoscopy	RRM
Case 5	41	BRCA2	91	10	4	Negative	Negative	Laparoscopy	Menopausal symptoms
Case 6	52	BRCA2	76	12	6	Negative	Negative	Laparotomy	Cervical herniation
Case 7	39	BRCA1	118	124	6	Negative	Negative	Laparoscopy →Laparotomy	Adhesions due to previous abdominal surgery; menopausal symptoms
Case 8	39	BRCA2	73	5	4	Negative	Negative	Laparoscopy	
Case 9	43	BRCA2	78	5	4	Negative	Negative	Laparoscopy	
Case 10	72	BRCA2	87	5	4	Negative	Negative	Laparoscopy	

**Figure 1:**
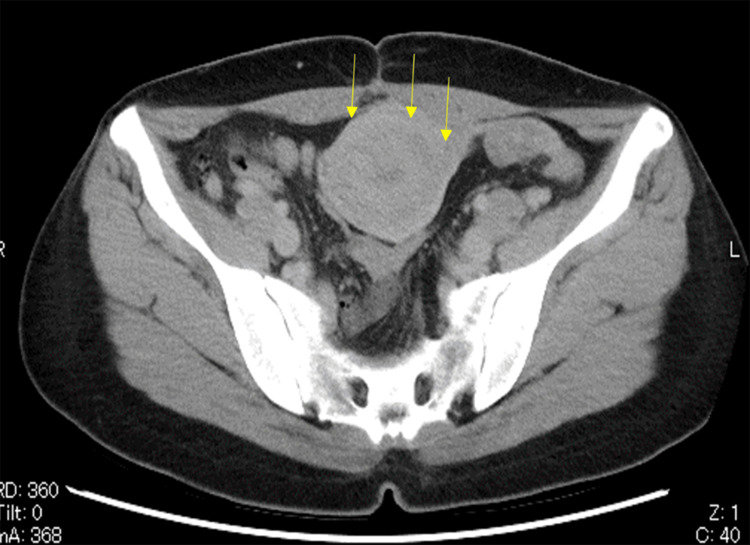
Computed tomography scan findings (Case 7) Findings suggestive of adhesions between the abdominal wall and uterus (arrow).

**Figure 2:**
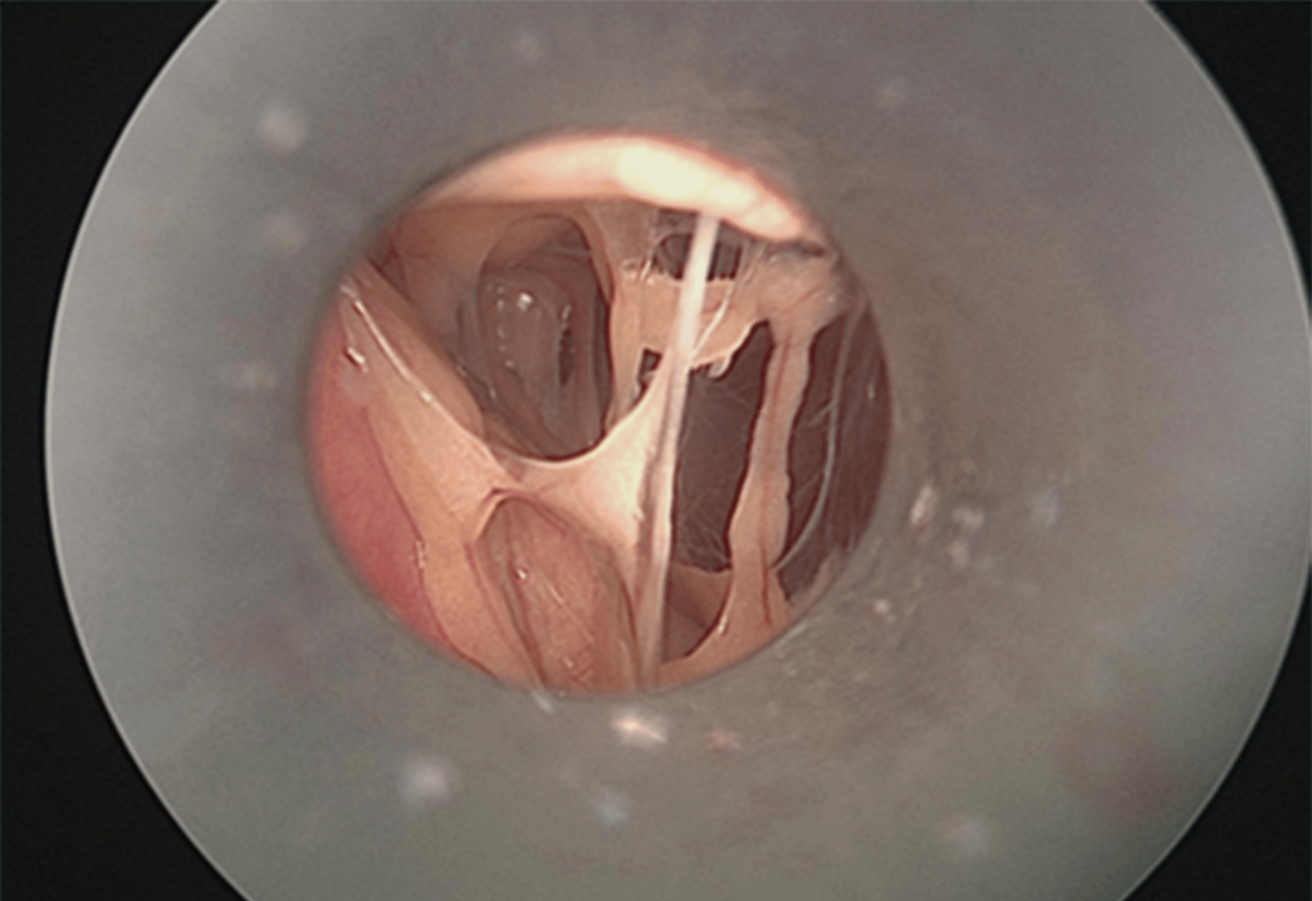
Intraperitoneal findings from the umbilicus first trocar, adhesion between the abdominal wall and omentum (Case 7) are noted.

**Figure 3:**
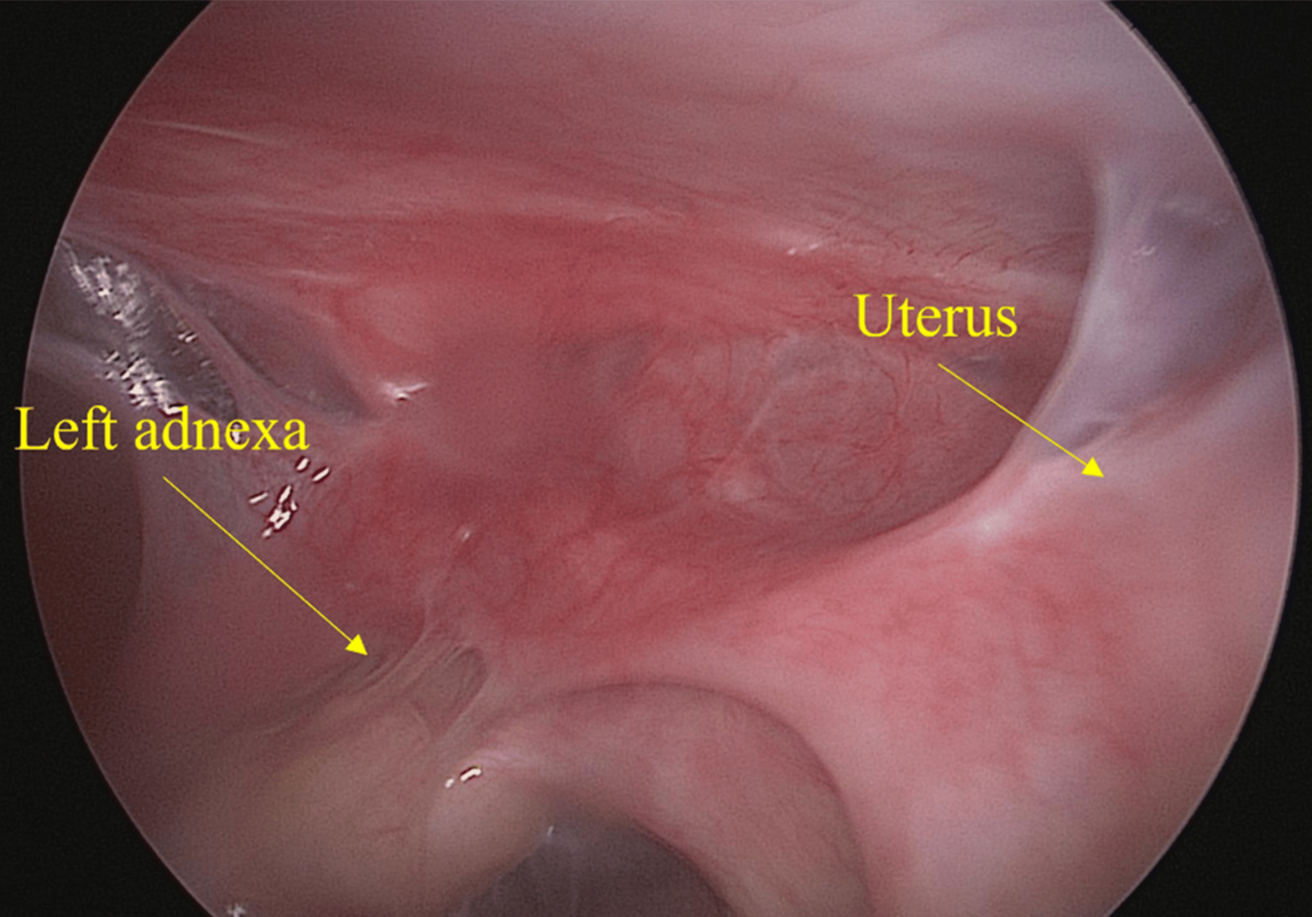
Intraperitoneal findings from the umbilicus first trocar, adhesions between the left appendage and the uterus (Case 7) are noted.

Case 6 had a cervical hernia, and in her case, endotracheal intubation was not possible. Therefore, RRSO was performed by laparotomy under spinal anesthesia and epidural anesthesia. Since the operation for Case 6 was performed by a midline incision from the lower abdomen, the visualization of the epigastric and intra-abdominal lumen was limited compared to the laparoscopic case. In addition, since Case 7 had undergone three cesarean sections, preoperative abdominal ultrasonography and CT (Figure 1) were taken, strongly suggesting intra-abdominal adhesions. A laparoscope was inserted through the umbilical cord to observe the intraperitoneal cavity and showed adhesions involving the abdominal wall, omentum, uterus, and appendages (Figures 2, 3). Given the number of adhesions present, it was determined that it would be difficult to insert a port into the pelvis. Therefore, RRSO was performed after laparoscopic evaluation of the entire intraperitoneal cavity. 

## Discussion

The preferred surgical procedure for RRSO is the minimally invasive approach, especially in consideration of removing healthy organs [9]. At our hospital, laparoscopic surgery tends to be more useful than laparotomy in terms of operative time, operative blood loss, and length of hospital stay. However, it is harder to visualize the upper abdomen and abdominal cavity during RRSO using a midline lower abdominal incision. We believe, therefore, that RRSO can achieve its original surgical purpose laparoscopically; however, in patients with a history of laparotomic surgery, if intraperitoneal adhesions are strongly suspected during the preoperative examination, it is important to determine where the first trocar can be safely inserted. In cases in which adhesions around the umbilicus are suspected based on previous surgery or preoperative diagnostic imaging, it is recommended that the first puncture be made at Palmer’s point (3 cm below the lower edge of the costal arch on the midclavicular line) [13].

It is necessary to monitor the progress of the patient after RRSO for the development of peritoneal cancer, treatment of menopausal symptoms after bilateral adnexal oophorectomy, and ongoing surveillance for HBOC. High-grade serous carcinoma of the ovaries is thought to originate from STIC in the fimbria of the fallopian tube [14]. There is no screening method for STIC, and the diagnosis can only be confirmed after adnexal resection. The detection rate of STIC after RRSO was reported to be 2.8% in an existing meta-analysis [15]. Furthermore, according to a meta-analysis published in 2022, the risk of developing peritoneal cancer after RRSO is 10.5% after five years and 27.5% after 10 years in patients with STIC. In contrast, in patients without STIC, the overall survival rates were 0.3% after five years and 0.9% after 10 years [10]. Based on these results, we feel that the postoperative follow-up period should be determined for each patient, based on the presence or absence of STIC during post-RRSO patient management. The 2019 NCCN Guidelines recommend follow-ups every two to four months for the first two years after ovarian cancer surgery, every six months for the next three years, and annually from the fifth year onwards [16]. European Society for Medical Oncology (ESMO) guidelines define that gynecological screening is not necessary after RRSO [17]. However; the treatment of STIC has not yet been finalized, we feel that patients with STIC should be followed up in the same manner as those with ovarian cancer, taking into account the incidence of postoperative peritoneal cancer. Currently, there is no treatment to reduce the incidence of peritoneal cancer after RRSO, so we believe that patients at risk for peritoneal cancer who have STIC should be carefully monitored.

Despite the effectiveness of premenopausal RRSO in reducing the risk of ovarian cancer and improving mortality in high-risk women, many patients experience troublesome menopausal symptoms (hot flashes, sweating, etc.), indicating that oophorectomy may have adverse long-term health consequences. In these cases, clinicians should focus on symptom relief by managing vasomotor, sleep, mood, sexual, and genitourinary symptoms to optimize long-term bone and cardiovascular health [18]. Kampo medicines are effective for menopausal symptoms such as sleep disorders and sweating. In Japan, Kampo medicines are commonly used for cases where hormone replacement therapy (HRT) is not possible, such as breast cancer [19]. Other treatments include soy isoflavones, known as phytoestrogens, which can help relieve hot flashes [20]. Kampo and soy isoflavones can be administered to breast cancer patients, and in this study, they were administered to patients who experienced sweating and hot flashes, and their symptoms improved.

The ESMO guidelines recommend annual breast screening with contrast-enhanced magnetic resonance imaging (MRI) in patients with HBOC [17]. Therefore, patients who have undergone surgical treatment for breast cancer but not contralateral prophylactic mastectomy should also undergo routine surveillance with contrast-enhanced breast MRI to ensure the early detection of breast cancer. They also report that patients who undergo RRSO should be informed of the short- and long-term health effects of early menopause. Some data suggest that HRT is safe, but recent studies suggest this may be true for women up to age 45. However, going beyond that may increase your risk of breast cancer, so you may be offered short-term HRT after RRSO. The limitations and risks of HRT should be clearly communicated, and while it reduces menopausal symptoms and the risk of osteoporosis, its benefits in cardiovascular and cognitive health remain controversial [17].

**Figure 4:**
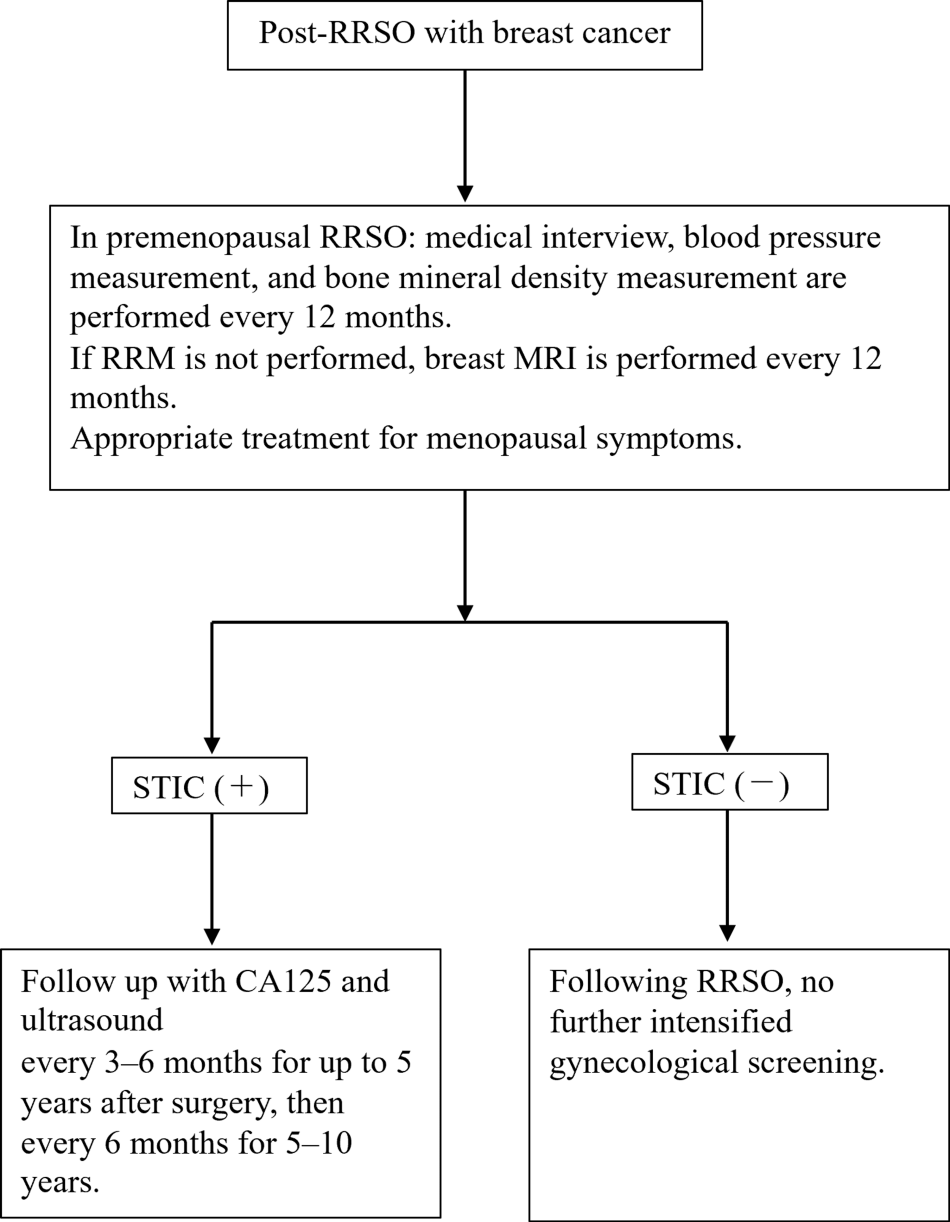
Guidelines for the postoperative management of RRSO at our hospital (based on the literature review) RRSO: risk-reducing salpingo-oophorectomy; STIC: serous tubal intraepithelial carcinoma; CA125: cancer antigen 125 This figure has been created by the authors and adapted from [16, 17].

We examined relevant literature, giving consideration to the appropriate management of patients after RRSO, and created a flowchart (Figure 4) to guide patient follow-up. In brief, for premenopausal patients with RRSO, medical interviews, blood pressure measurements, and bone mineral density measurements should be performed every 12 months. Furthermore, if a risk-reducing mastectomy is not performed, a breast MRI with contrast should be performed every 12 months. Menopausal symptoms should be treated with drug therapy.

To monitor for the development of peritoneal cancer, the length of the follow-up interval should be determined based on the presence or absence of STIC. In cases in which STIC is observed, the incidence of peritoneal cancer is significantly higher; therefore, we feel it is appropriate to monitor patient progression using transvaginal ultrasound and cancer antigen 125 (CA125), similar to ovarian cancer. In contrast, in cases in which STIC is not observed, the incidence of peritoneal cancer is low; therefore, we feel following RRSO, no further intensified gynecological screening.

This study was a retrospective study conducted at a single institution, with a small number of cases and limited statistical power. The small number of cases is related to the small number of genetic counseling sessions, and we believe that the first step is to increase the number of genetic counseling sessions. We would like to increase the number of cases at our own facility, collaborate with other medical institutions, and increase the number of cases. We would like to further examine the comparison of laparoscopy and laparotomy and the management after RRSO.

## Conclusions

While it is difficult to observe the upper abdomen during laparotomic RRSO, laparoscopy allows for the visualization of the entire abdominal cavity and tends to shorten the operative time compared with laparotomy; therefore, laparoscopic surgery is considered beneficial in that regard. The incidence of peritoneal cancer is higher in patients in whom STIC is observed than in those in whom it is not; therefore, it is necessary to individualize the intervals and duration of follow-up based on the presence or absence of STIC. Appropriate post-RRSO patient management, therefore, requires the consideration of the presence or absence of peritoneal cancer, surveillance of HBOC, and management of premature symptoms of menopause.
